# Supplementary figures and images for: Human Cerebrospinal Fluid Sample Preparation and Annotation for Integrated Lipidomics and Metabolomics Profiling Studies
Source: Mol Neurobiol. 2023 Oct 16;61(4):2021–32. doi: 10.1007/s12035-023-03666-4 (PMC10973045; doi:10.1007/s12035-023-03666-4)

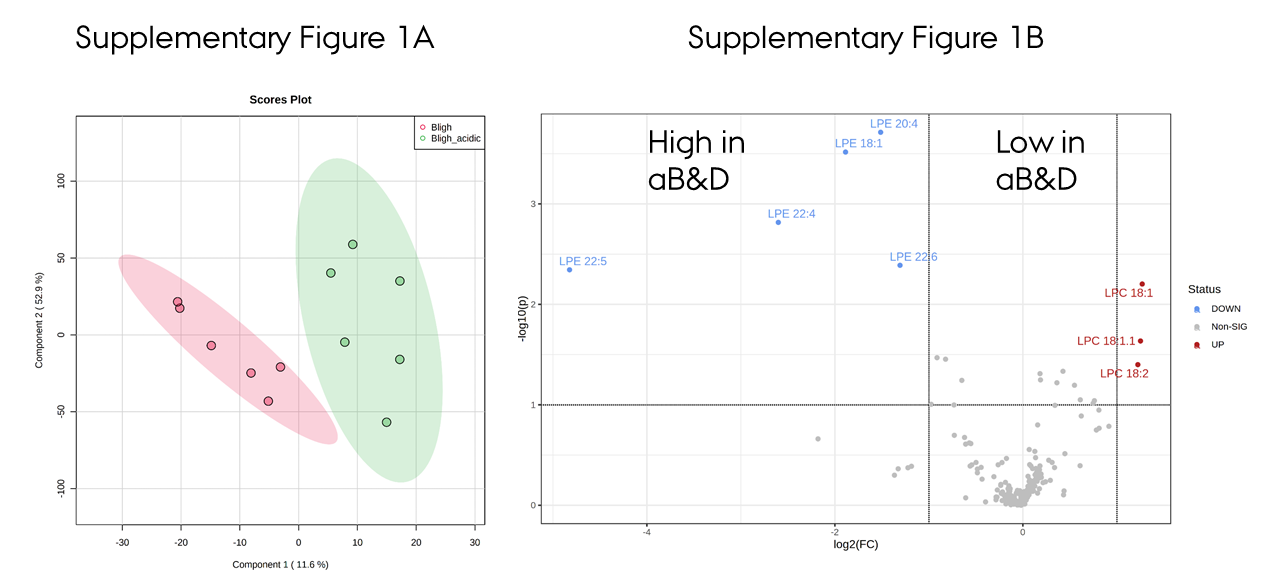

Supplement: Supplementary file 1 — Figure S1 (TIF 198 kb) [file 12035_2023_3666_MOESM1_ESM.tif]
