# Supplementary material for: Human Cerebrospinal Fluid Sample Preparation and Annotation for Integrated Lipidomics and Metabolomics Profiling Studies
Source: Mol Neurobiol. 2023 Oct 16;61(4):2021–32. doi: 10.1007/s12035-023-03666-4 (PMC10973045; doi:10.1007/s12035-023-03666-4)
Supplement: Supplementary file 7 — Table S5 (DOCX 17 kb) [file 12035_2023_3666_MOESM7_ESM.docx]

Table S5. The number of identified lipids from various studies, considering different extraction methods, LC-MS instruments, and CSF volume. Full names and abbreviations: Isopropanol (IPA), Methanol (MeOH), Bligh and Dyer (B&D), Quadrupole Time-of-Flight (QTOF).

| Extraction method | Instrument | CSF (µl) | Number of lipids | Reference |
| --- | --- | --- | --- | --- |
| Matyash | Direct infusion Q Exactive | 500 | 23 | [1] |
| Folch | Ion mobility QTOF | 75 | 38 | [2] |
| IPA | QTOF | 200 | 175 | [3] |
| Modified Folch | Q Exactive Orbitrap | 100 | 122 | [4] |
| Acidified MeOH | Q Exactive Orbitrap | 120 | 133 | [5] |
| Modified B&D | Q Exactive Orbitrap | 400 | 245 | [6] |

**References:**

1. Wood PL, Woltjer RL (2018) CSF Lipidomics Analysis: High-Resolution Mass Spectrometry Analytical Platform. In: Biomarkers for Alzheimer’s Disease Drug Development. Springer, pp 69-74

2. Reichl B, Eichelberg N, Freytag M, Gojo J, Peyrl A, Buchberger W (2020) Evaluation and optimization of common lipid extraction methods in cerebrospinal fluid samples. Journal of Chromatography B 1153:122271

3. Iriondo A, Tainta M, Saldias J, Arriba M, Ochoa B, Goñi FM, Martinez-Lage P, Abad-García B (2019) Isopropanol extraction for cerebrospinal fluid lipidomic profiling analysis. Talanta 195:619-627

4. Blasco H, Veyrat-Durebex C, Bocca C, Patin F, Vourc’h P, Kouassi Nzoughet J, Lenaers G, Andres CR, Simard G, Corcia P (2017) Lipidomics reveals cerebrospinal-fluid signatures of ALS. Scientific reports 7 (1):1-10

5. Saito K, Hattori K, Hidese S, Sasayama D, Miyakawa T, Matsumura R, Tatsumi M, Yokota Y, Ota M, Hori H (2021) Profiling of cerebrospinal fluid lipids and their relationship with plasma lipids in healthy humans. Metabolites 11 (5):268

6. Byeon SK, Madugundu AK, Jain AP, Bhat FA, Jung JH, Renuse S, Darrow J, Bakker A, Albert M, Moghekar A (2021) Cerebrospinal fluid lipidomics for biomarkers of Alzheimer's disease. Molecular Omics 17 (3):454-463
